# The effectiveness of propranolol, flunarizine, amitriptyline and botulinum toxin in vestibular migraine complaints and prophylaxis: a non-randomized controlled study

**DOI:** 10.1016/j.bjorl.2021.02.005

**Published:** 2021-03-07

**Authors:** Kemal Görür, Harun Gür, Onur İsmi, Cengiz Özcan, Yusuf Vayisoğlu

**Affiliations:** Mersin University, School of Medicine, Department of Otorhinolaryngology, Mersin, Turkey

**Keywords:** Vestibular migraine, Vestibular migraine treatment, Botulinum toxin

## Abstract

**Introduction:**

Vestibular migraine is the most common cause of spontaneous episodic vertigo in adult patients and the second most common cause of vertigo in patients of all ages.

**Objective:**

To assess the effectiveness of oral medication type (propranolol, flunarizine, and amitriptyline) and botulinum toxin A application on vestibular symptoms, headache severity and attack frequency for vestibular migraine patients.

**Methods:**

Sixty patients with vestibular migraine were enrolled. Thirty patients received botulinum toxin A treatment (B+ group) in addition to the oral medication, whereas 30 patients received only oral medication (B− group). Headache severity was evaluated with Migraine Disability Assessment Scale and vertigo severity was evaluated with Dizziness Handicap Inventory scale. Vestibular migraine attack frequencies in the last three months were also evaluated.

**Results:**

There was a statistically significant decrement in mean Dizziness Handicap Inventory scores, Migraine Disability Assessment Scale scores and vertigo attack frequencies after treatment for all patients, B+ and B− group patients (*p* < 0.001 for all). The mean Migraine Disability Assessment Scale score gains (*p* < 0.001) and vertigo attack frequency gains (*p* =  0.003) were significantly higher in the B+ patients than B− patients.

**Conclusions:**

Both B+ and B− group patients exhibited significant improvement in vestibular migraine attack frequencies, Dizziness Handicap Inventory score and Migraine Disability Assessment Scale score values. However, botulinum toxin A application had a more pronounced effect for Migraine Disability Assessment Scale score gain and vestibular migraine attack frequency values, but not for Dizziness Handicap Inventory score gain values. Thus, botulinum toxin A application should be considered for vestibular migraine patients whose headache severity degrees are more profound. The oral medication type (propranolol, flunarizine or amitriptyline) did not differ in influencing the vestibular migraine attack frequency, Dizziness Handicap Inventory score gain and Migraine Disability Assessment Scale score gain values.

## Introduction

Vestibular migraine (VM) is the most common cause of spontaneous episodic vertigo in the adult patients and the second most common cause of vertigo in patients of every age.^1^, ^2^, ^3^ Patients with VM commonly are admitted to vertigo clinics with dizziness and vertigo complaints, however there are no confirmatory diagnostic symptoms or findings. Therefore, these patients are frequently underdiagnosed. Dizziness and episodic vertigo can occur spontaneously or by head movements, positional changes, or visual stimuli. These attacks generally can be stimulated by stress, sleep disturbances, dehydration, menstruation, or certain foods.^4^ Occipital or hemi-cranial headache commonly accompanies the vertigo.^5^ VM has high prevalence for disabling situations; it decreases quality of life and labor force. Therefore, it needs certain diagnosis and effective treatment options.^6^ Neuhauser et al., have proposed the diagnostic criterion for definite and probable VM.^7^ Eleven years later these criteria were revised by the Barany Society and the International Headache Society in a consensus statement.^8^ The diagnosis is made according to the clinical description of the complaints and the symptoms reported by the patients. Additionally, other potential secondary causes must be excluded by appropriate investigation. The underlying causes of VM pathophysiology is not completely understood. The current proposed hypotheses are the same as migraine. It has been reported that reciprocal connections between the brainstem vestibular nuclei and the structures that modulate trigeminal nociceptive inputs may be involved in the pathogenesis of VM.^6^ Serotonin, noradrenalin, and dopamine, etc., may be involved in the pathogenesis of VM. During treatment of patients with VM these neurotransmitters may be regarded as options.^9^ Due to the fact that there are no evidence-based standardized treatment protocols, vestibular rehabilitation was used as a prophylactic and complementary treatment. Currently, several commonly used antimigraine pharmacological agents were adapted to treatment of VM. These are beta-blockers (e.g., propranolol, bisoprolol, metoprolol), calcium channel blockers (e.g., verapamil, amlodipine, flunarizine, cinnarizine), antiepileptic drugs (e.g., valproic acid, lamotrigine) and tricyclic antidepressants (e.g., amitriptyline, nortriptyline).^6^ Unfortunately, the effectiveness of these agents is often unsatisfactory. Recently, it was reported that only Botulinum toxin A (BTA) and topiramate have demonstrated efficacy in chronic migraine.^10^ In this study, we compared the effectiveness of propranolol, amitriptyline, flunarizine and BTA injection for VM prophylaxis regarding vertiginous symptoms and headache complaints.

## Methods

### Study design

This outpatient, open-label, prospective, non-randomized controlled trial with parallel groups was conducted at our Otolaryngology Department. Between January 2020 and September 2020 sixty of seventy-four patients were enrolled. Local ethics committee approval (approval number: 2020/386) and written informed consent from all patients were obtained before the initiation of the study.

### Subjects

All adult patients with VM criteria, as defined by the Barany Society and the International Headache Society were enrolled to the study.^11^ After the neurological and neurotological examinations, temporal bone magnetic resonance imaging (MRI), audiometric evaluation, videonystagmography, high-frequency headshake test, video head impulse test and bi-thermal caloric testing were performed to exclude other vestibular or neurological disorders. All patients who had exclusion criteria were excluded before randomization.

### Exclusion criteria

1 Patients with a known history of allergic reactions to propranolol, flunarizine, amitriptyline or botulinum toxin.
2 Patients who had no typical migrainous headache.
3 Female patients who were pregnant, planning for pregnancy or breast feeding.
4 Patients with a significant illness or medical condition such as cancer, liver, or kidney failure.
5 Patients who had certain medical conditions that could interfere with propranolol, calcium canal blockers, tricyclic antidepressant or BTA.

### Interventions

After the initial diagnosis of VM, all patients were questioned about their agreement for BTA (Botox, 100 units/vial, Allergan, UK) injection in addition to the oral medication therapy with one of the oral medication types (propranolol, amitriptyline or flunarizine). The patients who accepted BTA injection were taken into the group B+ and the patients who refused the BTA treatment were taken into the group B−. These two groups were again divided into three subgroups according to prescription of one of the three medications (propranolol, flunarizine or amitriptyline) according to their symptoms and medical conditions. Patients in two groups (group B+ and group B−) received propranolol (group B+P and group B–P), flunarizine (group B+F and group B–F) and amitriptyline (group B+A and group B—A). Patients who had depressive and sleep disturbances symptoms were prescribed amitriptyline. Propranolol was given for patients having palpitation and pulse rate over 80 min. Flunarizine was given to young and slim patients. Totally 155 units of BTA were injected with 8 mm × 30-gauge needle to the frontalis, corrugators, procerus, occipitalis, temporalis, trapezius and paraspinal muscle group in group B+.^12^, ^13^ Patients in the group B+P and group B–P received propranolol at a flexible dose of 20–80 mg, with an increasing dosage starting at 20 mg orally in the morning and 20 mg in the evening for a week, followed by 40 mg in the morning and 40 mg in the evening for 3 months. Thus, the total dose was up to 80 mg daily. For patients in group B+F and group B–F, flunarizine was started at 5 mg in the evening for a week and followed by 10 mg in the evening for 3 months. Thus, the total dose was up to 10 mg per day. Patients in group B+A and group B—A received amitriptyline 25–75 mg at evening for 3 months. The same diet modification and foods restriction were suggested to all patients. Life habits changing, restriction of some foods (caffeine, alcohol), regulation of sleep pattern, physical activity (walking for a 50 min per day) and vestibular rehabilitation (VR) (rotate the head from 30 to 180 degrees with opened eyes) were suggested to all of the study group patients.

### Assessment

The primary outcome was to compare the effectiveness of BTA, propranolol, flunarizine and amitriptyline for the reduction of vestibular symptoms and relief of the headache in VM patients. To evaluate the effects of the treatment on the vestibular symptoms, the Dizziness Handicap Inventory (DHI) and the number of vertiginous attacks at baseline and at third months of the treatment were evaluated.^14^ As mentioned; DHI scores were scaled between 0 and 100 points for each patient before and three months after the treatment. The subscales of the inventory including functional, emotional, and physical subscales were also counted for each patient.^14^ The number of vestibular attacks was evaluated for the number of vertiginous attacks in three months before and three months after the treatment. The Barany Society and the International Headache Society consensus statement diagnostic criteria were used for probable diagnosis of VM.^8^ Thus, vestibular symptoms such as spontaneous, positional, visual induced and head motion-induced vertigo were assessed at the first, second and at third months. A secondary outcome was to assess the effect of treatment on the headache. The Migraine Disability Assessment (MIDAS) questionnaire was used to quantify the severity of headache before and three months after the treatment.^15^ Additionally, to compare the effects of botulinum toxin injection and oral medication type on the complaints of VM, MIDAS score gains, DHI score gains and vertigo frequency gains were also measured by subtracting the post-treatment values from the initial values.

### Followup

After starting treatments, all patients were invited to the clinic biweekly for the evaluation of adverse effects and to be certain patient compliance with the drug dosage. All patients were informed to record of any vertigo attacks, headache, and number of experienced vertigo attacks during the treatment. Additionally, adverse effects, reason for exclusion such as refusal to participate, intolerance to treatment, were recorded during patient visits.

### Statistical analysis

Statistical analysis was performed using SPSS version 24.0 (IBM SPSS, New York, USA, 2016). Data were shown as mean ± standard deviation for continuous variables and number of cases was given for categorical ones. Data were controlled for normal distribution using the Shapiro-Wilk test. Chi-Square test was used to compare the gender differences and number of patients according to the oral medication drug types between B+ and B− group patients. Paired sample *t*-test was employed for comparison of mean MIDAS scores, DHI scores and vertigo attack frequencies before and after treatment. In addition, Mann-Whitney *U* test was used to compare the mean age, MIDAS score gain, DHI score gain and vertigo attack frequency gain values between B+ and B− patients. One-way ANOVA test was used to compare the mean MIDAS score gain, DHI score gain and vertigo attack frequency gain values between the oral medication types. Multivariate linear regression was performed to assess the relative effect of independent variables (Botox application status and oral medication type) on MIDAS score gain, DHI score gain and vertigo attack frequency gain values. Correlation coefficient was used to assess the correlation between MIDAS score gain values and DHI score gain values; *p*-value of <0.05 was regarded as statistically significant.

## Results

Fourteen patients were excluded from the study since they did not attend the control visits or exhibited medical side effects (pulse decreasing <50 min, hypotension, fatigue sensation or daytime sleepiness). Four patients did not come to control visits, six patients did not tolerate propranolol, three patients did not tolerate flunarizine and one patient discontinued amitriptyline treatment. There were 60 patients in the study group. The mean age was 45.86 ± 15.25 (min = 18, max = 75). 48 (80%) of the patients were female and 12 (20%) were male. 30 (50%) patients were given BTA treatment additional to the oral medication therapy, while the other 30 patients received only oral medication treatment. 25 (83.3%) patients in the B+ group patients and 23 (76.6%) patients in the B− group patients were female. The mean of the ages of the patients was 42.33 ± 13.21 and 48.8 ± 16.29 in the B+ and B− group patients, respectively. The number of patients receiving amitriptyline treatment was both 10 (33.3%) in the B+ and B− group patients. The number of patients receiving propranolol treatment was 11 (36.6%) in the B+ group and 9 (30%) in the B− group. The number of patients receiving flunarizine treatment was 9 (30%) in the B+ and 11 (36.6%) B− group patients. There was no statistically significant difference between B+ and B-group patients regarding gender (*p* = 0.519), mean age (*p* =  0.081) and type of oral medication (*p* =  0.819) differences.

**Figure 1:**
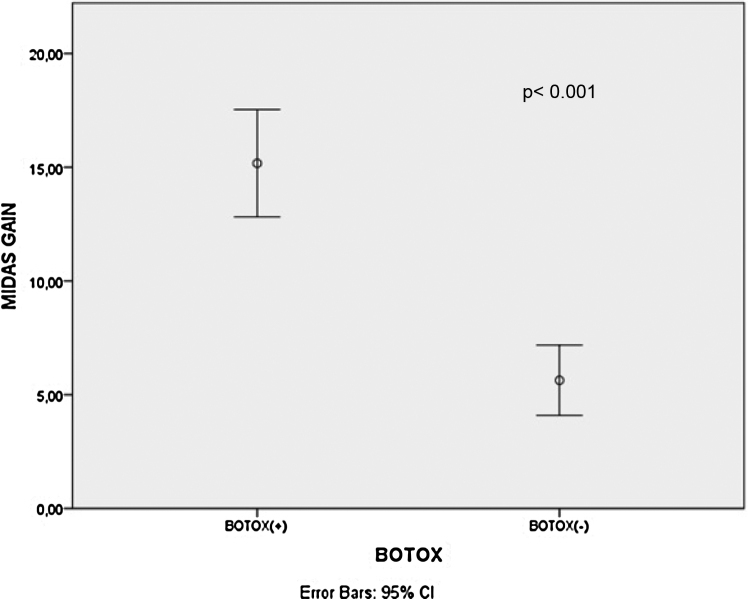
Comparison of MIDAS score gain values between Botulinum toxin applied and non-applied group patients. MIDAS, Migraine disability assessment scale; CI, Confident interval.

**Figure 2:**
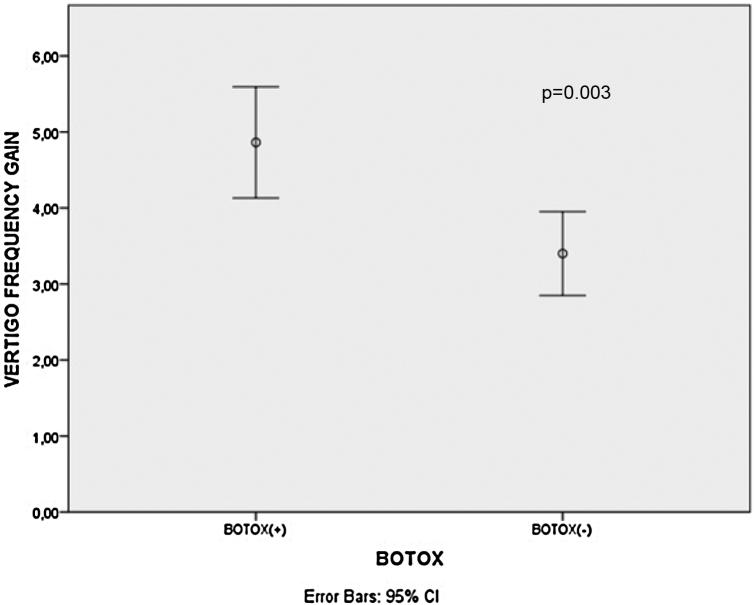
Comparison of vestibular migraine attack gain values between botulinum toxin applied and non-applied group patients. CI, Confident interval.

There was a statistically significant decrement in mean DHI scores, MIDAS scores and vertigo attack frequencies after treatment for all patients, B+ and B− group patients (*p* < 0.001 for all) as shown in Table 1. Additionally, the mean MIDAS score gains (*p* < 0.001) and vertigo attack frequency gains (*p* = 0.003) were significantly higher in the B+ patients than B− patients (Figure 1, Figure 2). However, there was no significant difference between B+ (40.9 ± 16.4) and B− (37.7 ± 18.1) group patients regarding the mean DHI score gain values (*p* = 0.466).

**Table 1:** Comparison of DHI scores, MIDAS scores and vertigo frequency values of the patients before and after the treatment.

Total DHI score	Pre-treatment (Mean ± SD)	Post-treatment (Mean ± SD)	*p*-value
All patients	60.8 ± 20.3	21.6 ± 13	< 0.001^a^
B+ patients	63.6 ± 18.3	22.7 ± 15.6	< 0.001^a^
B- patients	58.2 ± 22	20.5 ± 10.2	< 0.001^a^
DHI-P score			
All patients	17.7 ± 6.9	4.9 ± 4.1	< 0.001^a^
B+ patients	19.1 ± 6.4	6.1 ± 5	< 0.001^a^
B− patients	16.3 ± 7.2	3.7 ± 2.6	< 0.001^a^
DHI-E score			
All patients	19.2 ± 9.1	9.9 ± 6.2	< 0.001^a^
B+ patients	18.1 ± 9.4	8.9 ± 6.32	< 0.001^a^
B− patients	20.3 ± 8.9	10.9 ± 6.2	< 0.001^a^
DHI-F score			
All patients	24 ± 8.8	6.9 ± 5.2	< 0.001^a^
B+ patients	26.3 ± 7.6	7.8 ± 6.5	< 0.001^a^
B− patients	21.7 ± 9.4	6 ± 3.6	< 0.001^a^
MIDAS score			
All patients	15.7 ± 7.3	5.4 ± 4.3	< 0.001^*a^
B+ patients	18.8 ± 6.9	3.6 ± 3.5	< 0.001^a^
B− patients	12.8 ± 6.5	7.1 ± 4.4	< 0.001^a^
Vertigo frequency			
All patients	5.4 ± 1.7	1.3 ± 1	< 0.001^a^
B+ patients	6 ± 2	1.1 ± 1	< 0.001^a^
B− patients	4.9 ± 1.3	1.5 ± 1	< 0.001^a^

DHI, Dizziness handicap inventory, B+, Botulinum toxin applied patients; B-, Botulinum toxin non-applied patients; P, Physical; E, Emotional; F, Functional; MIDAS, Migraine disability assessment score.

a Statistically significant.

**Figure 3:**
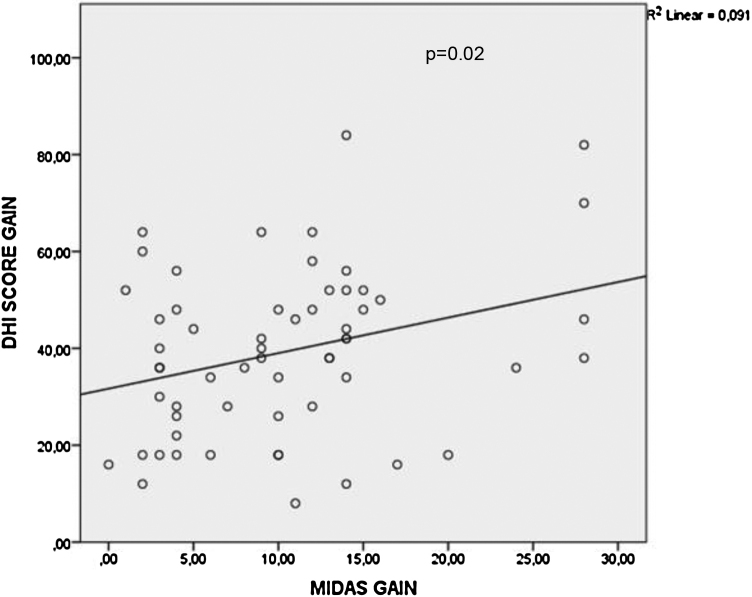
Correlation between MIDAS and DHI score gain values for vestibular migraine patients was demonstrated. MIDAS, Migraine disability assessment Scale; DHI, Dizziness handicap inventory.

It was observed that there was no statistically significant difference among the type of oral medications with respects to the mean MIDAS score gain (*p* = 0.667), DHI score gain (*p* = 0.4) and vertigo frequency gain (*p* = 0.388) values as shown in Table 2. When multivariate linear regression results were further analyzed, BTA application had statistically significant effect on mean MIDAS score gain values (*p* < 0.001) and vertigo frequency gain values (*p* = 0.003) compared to the oral medication type as shown in Table 3, Table 4. However, BTA application status (*p* = 0.488) or oral medication type (*p* = 0.383) did not have significant effects on DHI score gain values as shown in Table 5. Pearson correlation analysis demonstrated that there was a significant correlation between MIDAS score gain and DHI score gain values (r = 0.302, *p* = 0.02) (Fig. 3).

**Table 2:** Comparison of MIDAS score gain, DHI score gain and vertigo frequency gain values between oral medication types.

Medication type	Mean MIDAS score gain	*p*-value
Amitriptyline	9.15 ± 6.5	0.667
Propranolol	10.9 ± 6.7	
Flunarizine	11 ± 8.2	

Medication type	Mean DHI score gain	*p*-value
Amitriptyline	43.3 ± 16.8	0.4
Propranolol	36 ± 16	
Flunarizine	38.4 ± 18.9	

Medication type	Mean vertigo frequency gain	*p*-value
Amitriptyline	4.1 ± 2	0.388
Propranolol	4.6 ± 1.7	
Flunarizine	3.7 ± 1.9	

MIDAS, Migraine disability assessment; DHI, Dizziness handicap inventory.

**Table 3:** Multivariate linear regression results for the effect of independent variables on MIDAS score gain values.

Independent variable	β	Standard error	Significance
Botulinum toxin application status (applied vs. non-applied)	9.57	1.36	< 0.001^a^
Oral medication type (amitryptiline vs. propranolol vs. flunarizine)	1.057	0.837	0.212
Constant	3.52	1.92	0.073

β, Beta-coefficient; MIDAS, Migraine disability assessment.

a Statistically significant.

**Table 4:** Multivariate linear regression results for the effect of independent variables on vertigo frequency gain values.

Independent variable	β	Standard error	Significance
Botulinum toxin application status (applied vs. non-applied)	1.458	0.448	0.002^a^
Oral medication type (amitryptiline vs. propranolol vs. flunarizine)	−0.132	0.276	0.634
Constant	3.664	0.635	<0.001

β, Beta-coefficient.

a Statistically significant.

**Table 5:** Multivariate linear regression results for the effect of independent variables on DHI score gain values.

Independent variable	β	Standard error	Significance
Botulinum toxin application status (applied vs. non-applied)	3.146	4.51	0.488
Oral medication type (amitryptiline vs. propranolol vs. flunarizine)	−2.441	2.774	0.383
Constant	42.548	6.387	<0.001

β, Beta-coefficient; DHI, Dizziness handicap inventory.

During treatment of 60 patients, several side effects were reported by the patients as follows: the most commonly observed side effects was decrement of blood pressure and heart rate in 12 patients out of 20. Additionally, decrement of sexual pleasure and impotence was reported by two male patients during propranolol treatment. The most commonly reported side effects for flunarizine were somnolence and weight gain by the nine patients out of 20. Daytime sleepiness was reported by 5 patients out of 20. There were no patients exhibiting major side effects such as muscular weakness or skin reactions with regards to the BTA application. Six (20%) patients experienced minor pain at the BTA injection sites on the day of application.

## Discussion

In this study, we found that BTA application had a significant headache severity (MIDAS) score gain and attack frequency gain values for VM patients. On the other hand, BTA application did not decrease the vertigo attack severity (DHI) score gain significantly for these patients. Additionally, oral medication type (amitriptyline, propranolol, flunarizine) did not differ regarding the MIDAS score gain, DHI score gain and attack frequency gain values. Multivariate analysis results demonstrated that BTA application was the main determinant of MIDAS score gain and attack frequency gain values rather than the oral medication type for VM patients. There was a significant correlation between MIDAS score gain and DHI score gain values.

The main symptoms of patients with VM are episodic vertigo, headache, and imbalance. These symptoms may occur solitary or together during attacks and many patients are unaware that headaches originate from VM.^3^, ^16^

Several different treatment options were chosen randomly for the prophylaxis of VM by most of the studies.^4^, ^17^, ^18^, ^19^ Several studies have investigated the effectiveness of beta-blockers and anti-depressant drugs in VM prophylaxis. The overall effectiveness of propranolol was reported to be between 72% and 100%.^17^, ^18^, ^19^, ^20^ Salviz et al. reported that both propranolol and venlafaxine provide clinically relevant benefits for VM patients. Additionally, they found that venlafaxine is more effective than the propranolol for VM patients with severe depressive symptoms.^4^ Salmito et al. reported that amitriptyline, flunarizine, propranolol and topiramate improved the symptoms of patients with VM and there was no any significant difference among these drugs in the prophylaxis of VM regarding symptoms improving effects.^17^ Symptoms of VM, as a course of its nature, decreased or increased spontaneously during the treatment period, therefore, assessment of therapeutic response to treatment may be difficult during short period. To confer better reliability of therapeutic response, longer followup period may be needed. Hence, a longer duration of treatment and follow-up can increase the adverse effects of treatment. Therefore, we compared symptom scores of patients for three months before starting treatment and for three months after taking treatment.

Serious side effects can be seen during randomly chosen drugs in patients who had comorbidities. In our clinical experience, we thought that the drugs cannot be chosen randomly, they must be chosen according to patients’ medical conditions, presence of the comorbidities and symptoms. Dominguez-Duran et al. non-randomly prescribed one of the following drugs: acetazolamide, amitriptyline, flunarizine, propranolol or topiramate, and found the same effect of these drugs on reduction of symptoms and crises. They also suggested that drugs for prophylactic treatment of VM can be chosen regarding comorbidities of patients.^21^

BTA is another effective therapeutic option for treatment of chronic migraine.^22^, ^23^, ^24^ It was also approved by both the European Medicines Agency and the US Food and Drug Administration for the prophylaxis of chronic migraine. Recently, indications and dosage of the use of botulinum toxin in chronic migraine was also emphasized by European Headache Federation guidelines.^25^ Dodick et al. reported that only BTA and topiramate have demonstrated efficacy in chronic migraine.^10^ In this study, we also observed that BTA had a reasonable amount of effect in the improvement of VM symptoms, but this effect is more pronounced on headache scores than vertigo severity scores.

In order to increase tolerability and to decrease adverse effects of drugs (amitriptyline, propranolol and flunarizine) they were started at lower doses and increased gradually. However, ten patients did not tolerate our medications and were excluded from the study. Mild side effects of drugs during treatment were well tolerated by the patients and these side effects decreased one week after the initiation of the treatment. Better improvement on the headache symptom was seen by the BTA application in this study.

The pathophysiology of VM has not been established. Observations of patients during interictal period suggested that it is a central vestibular disorder, but peripheral vestibular reasons cannot be excluded.^26^ The mechanism of migraine aura and cerebellar symptoms can be explained by the cortical spreading depression theory.^27^ It has been reported that disrupting vestibular, visual, proprioceptive, and somatosensory afferent inputs originated from dysfunction of thalamocortical network.^28^ It has been theorized that the dizziness unrelated to headaches occurs from the release of neuropeptides (substance P, neurokinin A, and calcitonin gene-related peptide).^29^ Asymmetric neuropeptide releasing results in vertigo and increased motion sensitivity.^30^

Most of the patients with VM have vestibular-visual mismatch therefore vestibular rehabilitation (VR) is an effective nonmedical therapeutic option to treat dizziness and balance dysfunction and is based on central mechanisms of neuroplasticity. Vestibular rehabilitation facilitates vestibular compensation by adaptation, habituation, and substitution.^31^, ^32^ Despite vertigo control with prophylactic medication, VR may be indicated to resolve persistent imbalance.^3^ Therefore, some patients with persistent symptoms may benefit from VR. Vestibular suppressants are not suggested due to the concern that they affect the rate of central compensation.^33^ Changing of habits, restriction of some foods, regulation of sleep pattern, physical activity and VR were suggested to all of our patients. Therefore, effectiveness of these activity and restriction could not be evaluated due to lack of control group of only VR.

## Conclusion

In conclusion, sole oral medication therapy or combination of oral medication therapy with the BTA application improved both DHI scores and MIDAS scores and significantly decreased attack frequencies for vestibular migraine patients. Additionally, BTA application with the oral medication therapy had significant MIDAS score gain and attack frequency gain values as compared to the sole oral medication therapy. However, BTA application did not have any additional effect on the DHI score gain values as compared to the oral medication therapy. Thus, BTA application should be considered for VM patients whose headache severity degrees are more remarkable. VM patients should also be informed that BTA application in addition to the oral medication therapy does not exhibit significant decrement in the vertigo severity during VM attacks but decreases the attack frequency significantly as compared to the sole oral medication treatment modality. Oral medication type (propranolol, flunarizine or amitriptyline) did not differ regarding the VM attack frequency, DHI score gain and MIDAS score gain values.

## Funding

This research did not receive any specific grant from funding agencies in the public, commercial, or not-for-profit sectors.

## Ethics approval

This study was approved by local institutional review board of Mersin University (Date: 27.05.2020/nº 2020/386).

## Conflicts of interest

The authors declare no conflicts of interest.
